# Revisiting the physical activity paradox: the role of cardiorespiratory fitness in workers with high aerobic demands

**DOI:** 10.1177/14034948221151137

**Published:** 2023-02-02

**Authors:** Elin Ekblom-Bak, Björn Ekblom, Sofia Paulsson, Peter Wallin, Daniel Väisänen

**Affiliations:** 1 Department of Physical Activity and Health, The Swedish School of Sport and Health Sciences, Sweden; 2 HPI Health Profile Institute, Research Department, Danderyd, Sweden

**Keywords:** Cardiorespiratory fitness, occupational groups, occupational physical activity, physical activity paradox, workload

## Abstract

In contrast to leisure time physical activity, occupational physical activity may have adverse health effects—a phenomenon known as the “Physical activity paradox”. Characteristics such as long duration, low intensity, static and restricted movement, body position and insufficient recovery are possible explanations as to why physical activity in the occupational context may “wear one out” rather than provide health benefits. We emphasise the role of low cardiorespiratory fitness as a potential contributor to the physical activity paradox, and present data suggesting that only 25% to 50% of Swedish workers in occupations with higher aerobic demands may have “sufficient” cardiorespiratory fitness to maintain good health during their employment. More research is needed to fully understand the complexity of the role of other confounding factors when examining the relationship between cardiorespiratory fitness and occupational workload. However, we believe that there is an increasing need for general awareness amongst Swedish authorities, employees and employers of the potential health consequences of low levels of cardiorespiratory fitness, especially among workers with high occupational workloads. Importantly, when developing interventions targeting the working situation and/or cardiorespiratory fitness levels among workers, researchers should actively involve the relevant population in the design of the study in order to maximize the effect of the interventions on health outcomes.

## Introduction

Global guidelines on physical activity (PA) and sedentary behaviour recommend regular aerobic and muscle-strengthening activities, as well as limiting time spent sitting, for health promotion and disease prevention [1]. The recommendations are based primarily on evidence of the broad health benefits of leisure time PA and exercise. In contrast, no consensus has been reached as to the role of occupational PA (OPA) for health benefits. A phenomenon referred to as the “Physical activity paradox” has been proposed, implying that PA in the occupational context does not confer the same benefits as PA during leisure time, and may even induce detrimental health effects [2]. An umbrella review from 2018 found a lower risk of several cancer forms, cardiovascular disease and poor mental health with higher OPA [3], while data from a Norwegian study suggested that moderate to high OPA contributes to longevity in men [4]. Contrary to these findings, two meta-analyses found no association between OPA and overall cardiovascular mortality, as well as a non-significant increase in the risk of mortality from ischemic heart disease [5], and an increased risk of early mortality in men [6]. Methodological differences between the studies, and the influence of residual confounding associated with different lifestyles, environmental exposure and socioeconomic status between the occupational groups need to be more closely considered [7,8]. Efforts have been made to target the latter, where even after taking into consideration various lifestyle-, health-, living- and socioeconomic-related factors, the detrimental association between higher OPA and the incidence of cardiovascular disease and all-cause mortality remained [6,9,10].

## Mechanisms to explain the physical activity paradox

A next, crucial step to consider in the PA paradox discussion is possible mechanisms. Some suggestions have been made that are related to the general characteristics and context in which OPA is performed [2, 11]. While leisure time PA usually constitutes dynamic and unrestricted movement and body positions with short duration (30–60 min), higher intensity and sufficient recovery between sessions, as well as the activity often being chosen by the individual, OPA generally has the opposite characteristics. These include long duration (⩾8 h), lower/moderate intensities, static and restricted movement and body position, insufficient recovery and less autonomy of the activity performed. In total, these characteristics may increase the likelihood of “wearing the worker out” rather than providing health benefits. This has been elucidated in several cross-sectional studies [12,13]. Using 24-h blood pressure measurements, workers with high OPA had higher mean systolic blood pressure at work, at home and while sleeping. This was in contrast to workers with large amounts of leisure time exercise, where significantly lower mean systolic blood pressure over 24 h was seen [13]. Moreover, in approximately 13,000 participants, those engaging in high levels of leisure PA had lower odds ratios of having elevated levels of high-sensitivity C-reactive protein (marker of inflammation) [12]. On the contrary, participants with high levels of OPA had higher odds ratios, which further increased for those with high OPA and low leisure time PA.

## Cardiorespiratory fitness—an important factor for healthy employment?

Another possible explanation for the prevalence of adverse outcomes in workers with high OPA is insufficient cardiorespiratory fitness in relation to the aerobic demands of the occupation. Cardiovascular fitness, often assessed as maximal oxygen consumption (VO_2_max), is an independent predictor for several non-communicable diseases and premature mortality [14, 15]. A moderate-to-high VO_2_max is a prerequisite for aerobic endurance performance [16] and, as such, also for engaging in prolonged exercise activities and daily life activities [17]. VO_2_max is likewise crucial for the average, relative workload during the working day. Daily average workload in 497 blue-collar workers was measured and expressed as a percentage of heartrate reserve (interpretable as percentage of VO_2_max) [18]. Workers with higher fitness had lower average daily workloads, as well as less time spent above the recommended upper limit of average daily workload (~30% of heartrate reserve [19]). Translating this into possible adverse health effects, a study including blue-collar workers showed that more time spent above this recommended upper limit of average, relative work load was associated with lower heartrate variability and a higher average heartrate the following night [20]. Both these variables may indicate an imbalanced autonomic cardiac modulation in response to a high occupational workload, which could partly explain the higher incidence of cardiovascular disease reported in these occupational groups.

## Declining trend in cardiorespiratory fitness

We recently presented data from occupational health profile assessments in over 350,000 men and women from the Swedish working population that showed a decline in cardiovascular fitness over the last few decades [21]. This trend was confirmed in a systematic review including over 2.5 million adults from high- and upper-to-middle income countries [22]. The decline was more pronounced in men, younger age-groups [21,22], and in blue-collar or low-skilled occupations [23]. Prognostic analyses forecast a continued downward trend of cardiovascular fitness, especially in low-skilled white-collar and blue-collar occupations [23]. This may be counterintuitive, as many consider PA at work to be a “work-out”, while it often has too low intensity to improve cardiovascular fitness [18,24,25]. In addition, the long durations of PA at a high mean intensity during working hours may be sufficiently tiring for the individual, leading to less engagement in vigorous fitness-enhancing leisure-time PA—as often seen in individuals with heavy occupational loads [26,27].

## Are today’s workers fit for work?

It would be highly relevant to reveal the proportion of workers from different occupational groups who have “sufficient” cardiovascular fitness in relation to their work task. Unfortunately, no published data are available on this subject. However, to get an estimate, we used data from a recent publication on the average workload of different occupational groups [28], as well as data on estimated VO_2_max from the health profile assessments referred to above [21,23]. A randomly selected matched sample, concerning sex, age and self-reported workload (*N* = 50,000, 2015–2020), was drawn from the cohort of health profile assessments, and matched to the cohort with the average workload data. The same definitions of occupational groups (International Standard Classification of Occupations) were used.

**Figure 1.**
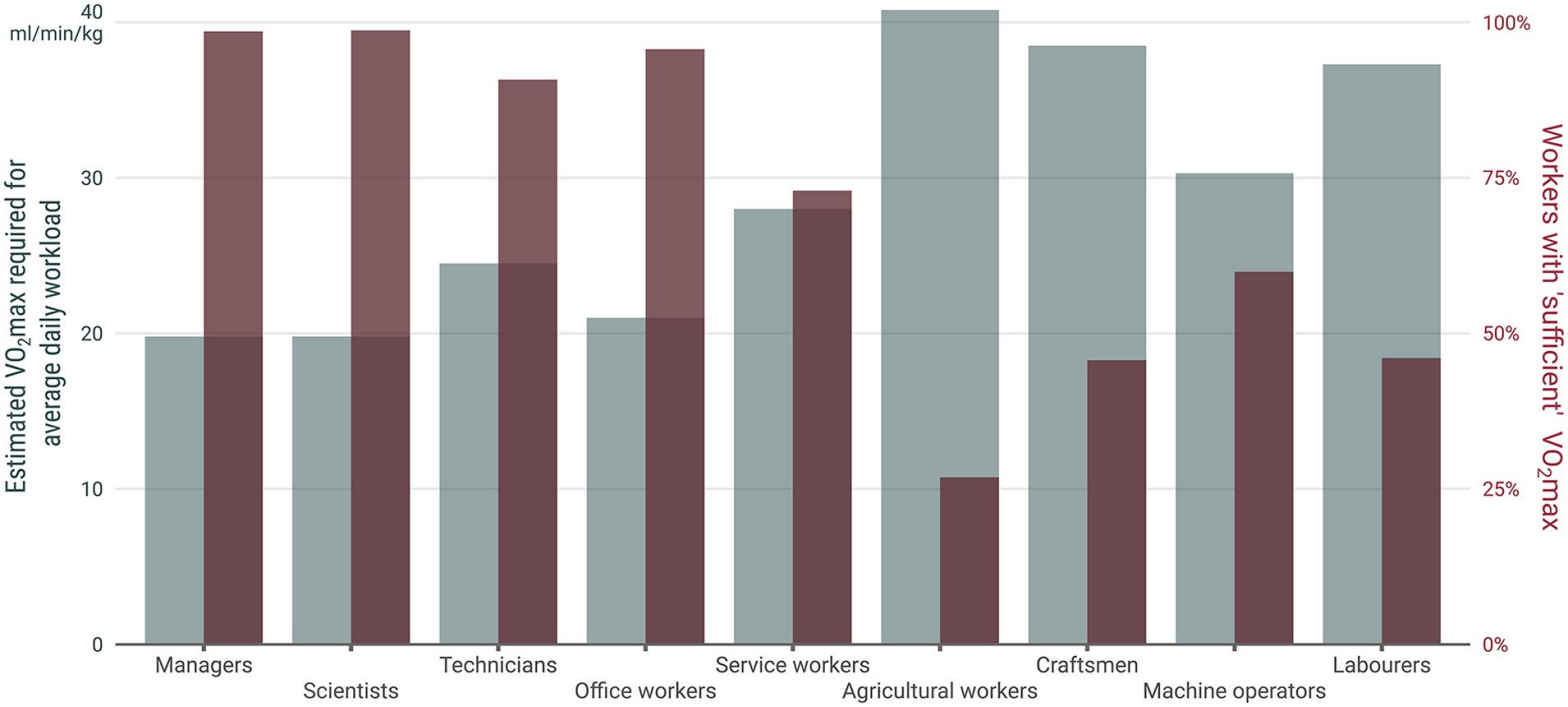
The estimated VO_2_max required for average daily workload not exceeding 30% of maximal capacity in different occupational groups, and the proportion of workers with “sufficient” VO_2_max in relation to their workload. The workload in each occupational group was reported by Brighenti-Zogg et al. [28] in metabolic equivalents (METs), which in turn was transformed into relative VO_2_max (ml min^−1^ kg^−1^) by multiplying the MET value with its conventional equivalent 3.5 ml min^−1^ kg^−1^. The estimated VO_2_max required was then calculated by dividing the average daily workload in ml min^−1^ kg^−1^ by 0.30. This proportion represents the recommended level (30% of VO2max) that average daily workload should not exceed [18] (Formula Whole = Part/Proportion). The average workload in percentage of VO_2_max was calculated for each participant by dividing the transformed workload in ml min^−1^ kg^−1^ by estimated VO_2_max in ml min^−1^ kg^−1^.

In Figure 1, we present the proportion of workers (right *y* axis) in different occupational groups (*x* axis) with “sufficient” VO_2_max levels in relation to estimated VO_2_max required for the average daily workload of each occupational group (left *y* axis). A “sufficient” VO_2_max was defined as having a VO_2_max level so that the average daily workload did not exceed 30% of VO_2_max [18].

A large majority (91%–99%) of workers in occupations with low workloads (managers, scientists, technicians and office workers) had a sufficient VO_2_max relative to their work tasks. However, among occupations with higher average workloads (agricultural workers, craftsmen, machine operators and labourers), only every fourth to every second employee had a sufficient VO_2_max. This indicates that an insufficient VO_2_max may be a contributor to the adverse health outcomes in these groups, which is in addition to the above-described characteristics of long duration, static and restricted movement and insufficient recovery associated with high OPA. This is of particular concern among the aging population. In a recent publication, older construction- and healthcare workers had lower cardiorespiratory fitness than their younger colleagues, regardless of similar or higher physical demands [25]. This warrants future research on how to also organize work in relation to age and aerobic capacity so as to enable sustainable employment.

## Conclusion, and what then?

Although many occupations have developed and become less physically demanding in the last few decades, a large part of the workforce still works in occupations with high aerobic demands. Although some thought-provoking research points to a potential PA paradox, further investigation is needed to confirm whether this is true, and for whom, and when high OPA may be detrimental. Based on basic principles of work physiology and previous epidemiological evidence on the importance of cardiovascular fitness for health and performance in general, the potentially negative role of low cardiorespiratory fitness should be a target for improving sustainable work participation. More research is warranted on the absolute and relative strain among different occupational groups, and to understand the complexity of the issue. However, in light of the large numbers of Swedish workers in our sample with “insufficient” cardiorespiratory fitness, it is a matter of urgency to create awareness of the issue, and for authorities and employees to develop preventative strategies. Using the workplace as an arena for improving the health of workers, as opposed to general community-based health-promoting interventions, has been suggested as a more effective strategy to reach and include groups that might otherwise be difficult to motivate. Importantly, when designing interventions targeting the working situation and/or cardiorespiratory fitness levels among workers, researchers should actively involve the relevant population in the design of the study in order to maximize the effect of the interventions on health outcomes [29]. Some promising research and initiatives have already been suggested [30–32], but more research is required before these can be implemented on a larger scale.

## References

[1] Bull FC Al-Ansari SS Biddle S , et al. World Health Organization 2020 guidelines on physical activity and sedentary behaviour. Br J Sports Med 2020;54:1451-62. 10.1136/bjsports-2020-102955 PMC7719906 33239350

[2] Holtermann A Krause N van der Beek AJ , et al. The physical activity paradox: six reasons why occupational physical activity (OPA) does not confer the cardiovascular health benefits that leisure time physical activity does. Br J Sports Med 2018;52:149-50. 10.1136/bjsports-2017-097965 28798040

[3] Cillekens B Lang M van Mechelen W , et al. How does occupational physical activity influence health? An umbrella review of 23 health outcomes across 158 observational studies. Br J Sports Med 2020;54:1474-81. 10.1136/bjsports-2020-102587 33239353

[4] Dalene KE Tarp J Selmer RM , et al. Occupational physical activity and longevity in working men and women in Norway: a prospective cohort study. Lancet Public Health 2021;6:e386-95. 10.1016/S2468-2667(21)00032-3 33932334

[5] Cillekens B Huysmans MA Holtermann A , et al. Physical activity at work may not be health enhancing. A systematic review with meta-analysis on the association between occupational physical activity and cardiovascular disease mortality covering 23 studies with 655 892 participants. Scand J Work Environ Health 2022;48:86-98. 34656067 10.5271/sjweh.3993 PMC9045238

[6] Coenen P Huysmans MA Holtermann A , et al. Do highly physically active workers die early? A systematic review with meta-analysis of data from 193 696 participants. Br J Sports Med 2018;52:1320-6. 10.1136/bjsports-2017-098540 29760168

[7] Vaisanen D Kallings LV Andersson G , et al. Lifestyle-associated health risk indicators across a wide range of occupational groups: a cross-sectional analysis in 72,855 workers. BMC Public Health 2020;20:1656. 33148214 10.1186/s12889-020-09755-6 PMC7641800

[8] Clougherty JE Souza K Cullen MR . Work and its role in shaping the social gradient in health. Ann N Y Acad Sci 2010;1186:102-24. 10.1111/j.1749-6632.2009.05338.x PMC3704567 20201870

[9] Smith P Ma H Glazier RH , et al. The relationship between occupational standing and sitting and incident heart disease over a 12-year period in Ontario, Canada. Am J Epidemiol 2018;187:27-33. 29020132 10.1093/aje/kwx298 PMC5860480

[10] Holtermann A Schnohr P Nordestgaard BG Marott JL . The physical activity paradox in cardiovascular disease and all-cause mortality: the contemporary Copenhagen General Population Study with 104 046 adults. Eur Heart J 2021;42:1499-511. 10.1093/eurheartj/ehab087 PMC8046503 33831954

[11] Halle M Heitkamp M . Prevention of cardiovascular disease: does ‘every step counts’ apply for occupational work? Eur Heart J 2021;42:1512-5. 10.1093/eurheartj/ehab105 33831947

[12] Lee J Kim HR Jang TW , et al. Occupational physical activity, not leisure-time physical activity, is associated with increased high-sensitivity C reactive protein levels. Occup Environ Med 2021;78:86-91. 32912859 10.1136/oemed-2020-106753

[13] Clays E De Bacquer D Van Herck K , et al. Occupational and leisure time physical activity in contrasting relation to ambulatory blood pressure. BMC Public Health 2012;12:1002. 23164344 10.1186/1471-2458-12-1002 PMC3551777

[14] Kodama S Saito K Tanaka S , et al. Cardiorespiratory fitness as a quantitative predictor of all-cause mortality and cardiovascular events in healthy men and women: a meta-analysis. JAMA 2009;301(19):2024-35. 10.1001/jama.2009.681 19454641

[15] Laukkanen JA Isiozor NM Kunutsor SK . Objectively assessed cardiorespiratory fitness and all-cause mortality risk: an updated meta-analysis of 37 cohort studies involving 2,258,029 participants. Mayo Clin Proc 2022;97:1054-73. 10.1016/j.mayocp.2022.02.029 35562197

[16] Bassett DR Jr Howley ET . Limiting factors for maximum oxygen uptake and determinants of endurance performance. Med Sci Sports Exerc 2000;32:70-84. 10647532 10.1097/00005768-200001000-00012

[17] Ainsworth BE Haskell WL Herrmann SD , et al. 2011 Compendium of Physical Activities: a second update of codes and MET values. Med Sci Sports Exerc 2011;43:1575-81. 10.1249/MSS.0b013e31821ece12 21681120

[18] Stevens ML Crowley P Holtermann A , et al. Cardiorespiratory fitness, occupational aerobic workload and age: workplace measurements among blue-collar workers. Int Arch Occup Environ Health 2021;94:503-13. 10.1007/s00420-020-01596-5 PMC8032632 33161441

[19] Wu HC Wang MJ . Relationship between maximum acceptable work time and physical workload. Ergonomics 2002;45:280-9. 10.1080/00140130210123499 12028725

[20] Korshoj M Lund Rasmussen C de Oliveira Sato T , et al. Heart rate during work and heart rate variability during the following night: a day-by-day investigation on the physical activity paradox among blue-collar workers. Scand J Work Environ Health 2021;47:387-94. 10.5271/sjweh.3965 PMC8259705 33929548

[21] Ekblom-Bak E Ekblom O Andersson G , et al. Decline in cardiorespiratory fitness in the Swedish working force between 1995 and 2017. Scand J Med Sci Sports 2019;29:232-9. 10.1111/sms.13328 PMC7379642 30351472

[22] Lamoureux NR Fitzgerald JS Norton KI , et al. Temporal trends in the cardiorespiratory fitness of 2,525,827 adults between 1967 and 2016: a systematic review. Sports Med 2019;49:41-55. 30390202 10.1007/s40279-018-1017-y

[23] Väisänen D Kallings LV Andersson G , et al. Cardiorespiratory fitness in occupational groups-trends over 20 years and future forecasts. Int J Environ Res Public Health 2021;18:8437. 34444184 10.3390/ijerph18168437 PMC8394663

[24] Korshoj M Krustrup P Jespersen T , et al. A 24-h assessment of physical activity and cardio-respiratory fitness among female hospital cleaners: a pilot study. Ergonomics 2013;56:935-43. 10.1080/00140139.2013.782427 23586528

[25] Merkus SL Lunde LK Koch M , et al. Physical capacity, occupational physical demands, and relative physical strain of older employees in construction and healthcare. Int Arch Occup Environ Health 2019;92:295-307. 30443711 10.1007/s00420-018-1377-5 PMC6420471

[26] Ekblom-Bak E Borjesson M Bergman F , et al. Accelerometer derived physical activity patterns in 27.890 middle-aged adults: the SCAPIS cohort study. Scand J Med Sci Sports 2022;32:866-80. 10.1111/sms.14131 PMC9302631 35080270

[27] Biswas A Dobson KG Gignac MAM , et al. Changes in work factors and concurrent changes in leisure time physical activity: a 12-year longitudinal analysis. Occup Environ Med 2020;77:309-15. 10.1136/oemed-2019-106158 32107318

[28] Brighenti-Zogg S Mundwiler J Schupbach U Dieterle T Wolfer DP Leuppi JD , et al. Physical workload and work capacity across occupational groups. PloS One 2016;11:e0154073. 10.1371/journal.pone.0154073 PMC4852946 27136206

[29] Cargo M Mercer SL . The value and challenges of participatory research: strengthening its practice. Annu Rev Public Health 2008;29:325-50. 10.1146/annurev.publhealth.29.091307.083824 18173388

[30] Lerche AF Mathiassen SE Rasmussen CL , et al. Development and implementation of ‘Just Right’ physical behavior in industrial work based on the Goldilocks work principle—a feasibility study. Int J Environ Res Public Health 2021;18:4707. 33925078 10.3390/ijerph18094707 PMC8125316

[31] Lerche AF Vilhelmsen M Schmidt KG , et al. Can childcare work be designed to promote high intensity physical activity for improved fitness and health? A proof of concept study of the Goldilocks Principle. Int J Environ Res Public Health 2020;17:7419. 33053791 10.3390/ijerph17207419 PMC7600739

[32] Naweed A Chapman J Vandelanotte C , et al. ’Just Right’ job design: a conceptual framework for sustainable work in rail driving using the Goldilocks work paradigm. Appl Ergon 2022;105:103806. 35772288 10.1016/j.apergo.2022.103806

